# Clinical pharmacist-guided management of heparin-induced thrombocytopenia with thrombosis after hip hemiarthroplasty: a case report

**DOI:** 10.3389/fcvm.2026.1897675

**Published:** 2026-07-31

**Authors:** Qinan Yin, Lizhu Han, Yuan Bian, Yujie Song, Youjin Huang

**Affiliations:** 1 Department of Pharmacy, Personalized Drug Research and Therapy Key Laboratory of Sichuan Province, Sichuan Provincial People's Hospital, School of Medicine, University of Electronic Science and Technology of China, Chengdu, China; 2 Clinical Trial Centre, Sichuan Academy of Medical Sciences & Sichuan Provincial People's Hospital, Chengdu, China; 3 Department of Vascular Surgery Center, Sichuan Academy of Medical Sciences & Sichuan Provincial People's Hospital, School of Medicine, University of Electronic Science and Technology of China, Chengdu, China

**Keywords:** analysis of cases, anticoagulant therapy, enoxaparin, fondaparinux, heparin-induced thrombocytopenia

## Abstract

**Objective:**

To describe the clinical management of heparin-induced thrombocytopenia with thrombosis (HITT) after orthopedic surgery and to highlight the role of clinical pharmacists in early recognition and individualized anticoagulant therapy.

**Methods:**

An 85-year-old woman developed HITT after right hip hemiarthroplasty and low molecular weight heparin thromboprophylaxis. Following the development of thrombocytopenia and new thrombotic events, clinical pharmacists participated in multidisciplinary evaluations, calculated the 4Ts score, recommended HIT antibody testing, and assisted in anticoagulant adjustment. Platelet count, D-dimer levels, renal function, and anti-PF4/heparin antibody levels were monitored throughout hospitalization.

**Results:**

The patient developed bilateral calf intermuscular venous thrombosis and multiple pulmonary emboli following orthopedic surgery. The 4Ts score was 6, and anti-PF4/heparin antibody testing was positive (5.7 U/mL; reference range, 0–1.0 U/mL), confirming the diagnosis of HITT. Enoxaparin was discontinued, and anticoagulation was switched to fondaparinux sodium. Platelet counts gradually recovered from 27 × 10^9^/L to 253 × 10^9^/L during follow-up, with no recurrent thrombosis or bleeding events reported during the available follow-up period.

**Conclusion:**

HITT after orthopedic surgery may be difficult to recognize because postoperative thrombocytopenia has multiple causes. This case highlights the importance of timely assessment of HIT probability and multidisciplinary collaboration. Clinical pharmacists may contribute to the early diagnosis, appropriate anticoagulant modification, and individualized management of patients with complex anticoagulation needs.

## 1 Introduction

Heparin-induced thrombocytopenia (HIT) is a rare but potentially life-threatening immune-mediated adverse reaction associated with heparin exposure. HIT is characterized by a significant reduction in platelet count accompanied by a paradoxical increase in thrombotic risk, with approximately one-third to one-half of affected patients developing HIT-associated thrombosis (HITT) (1–3). Although the incidence of HIT has decreased with the widespread use of low-molecular-weight heparin (LMWH), delayed recognition and inappropriate continuation of heparin therapy may result in severe thrombotic complications and adverse outcomes (2, 4).

Patients undergoing major orthopedic surgery are particularly vulnerable to venous thromboembolism (VTE) and HIT. Hip fracture surgery and joint replacement procedures are associated with a high baseline risk of postoperative VTE, and pharmacological thromboprophylaxis with LMWH or other anticoagulants is routinely recommended (5, 6). However, postoperative thrombocytopenia is common after orthopedic procedures, making it challenging to distinguish HIT from other causes of platelet decline. Therefore, careful monitoring of platelet trends, assessment of clinical probability, and timely adjustment of anticoagulant therapy are essential for the early diagnosis and effective management of HIT in this population (4, 7).

Clinical pharmacists may contribute significantly to the multidisciplinary management of HIT by identifying potential cases, facilitating diagnostic evaluations, and optimizing anticoagulant selection. However, detailed reports describing pharmacists' involvement in the recognition and individualized treatment of HITT after orthopedic surgery remain limited. Herein, we report a case of HITT occurring after hip hemiarthroplasty in an older patient. This case highlights the diagnostic challenges in distinguishing HIT from postoperative thrombocytopenia, the importance of timely pharmacist intervention in anticoagulant decision-making, and the individualized use of fondaparinux for managing HIT-associated thrombosis.

## 2 Case description

An 85-year-old woman (height, 158 cm; weight, 50 kg) was admitted on December 24, 2025, after a mechanical fall that resulted in acute right hip pain and impaired mobility of the right lower limb. Radiographic examination confirmed a displaced right femoral neck fracture, and thoracic computed tomography revealed suspected non-displaced fractures of the right third to sixth ribs. Lower extremity color Doppler ultrasonography revealed no evidence of deep vein thrombosis (DVT) upon admission. The initial diagnoses were right femoral neck fracture and suspected rib fractures.

Prophylactic anticoagulation with enoxaparin (3,000 IU subcutaneously once daily) was initiated after the patient's admission. The baseline platelet count was 109 × 10^9^/L on December 24. Analgesic therapy included tramadol hydrochloride (100 mg, orally, once daily). On December 26, the patient underwent right hip hemiarthroplasty under general anesthesia. Perioperative prophylactic antibiotics consisted of intravenous cefuroxime (1.5 g) administered 30 min before the incision. The estimated intraoperative blood loss was 100 mL, and tranexamic acid (1 g) was administered intra-articularly.

After surgery, the patient was transferred to the intensive care unit for postoperative monitoring and received intravenous oxycodone via patient-controlled analgesia, omeprazole (40 mg intravenously once daily), and enoxaparin (2,000 IU subcutaneously once daily). On December 27, her clinical condition stabilized, and she was transferred to the orthopedic ward. At that time, her platelet count was 86 × 10^9^/L. Analgesic therapy was adjusted to oral etoricoxib (120 mg once daily), and anticoagulation therapy was switched from prophylactic enoxaparin to apixaban (2.5 mg orally twice daily) for postoperative thromboprophylaxis after surgery.

On December 30 (postoperative day 4), the patient developed acute dyspnea, chest tightness, and altered mental status. Laboratory evaluation showed a marked elevation in D-dimer levels (7.86 mg/L; baseline, 3.53 mg/L) and hypoxemia (PaO_2_, 69.9 mmHg). Lower extremity Doppler ultrasonography confirmed bilateral calf intermuscular venous thrombosis, and pulmonary computed tomography angiography revealed multiple segmental and subsegmental pulmonary emboli. Apixaban was discontinued, and therapeutic anticoagulation with enoxaparin (4,000 IU subcutaneously every 12 h) was initiated according to the standard management approach for acute venous thromboembolism. At that time, HIT was not suspected.

On December 31, the platelet counts further decreased to 60 × 10^9^/L. Her serum creatinine level was 42.9 μmol/L, and the estimated creatinine clearance (CrCl) was 66.61 mL/min, indicating a preserved renal function. A clinical pharmacist consultation was requested because of the combination of progressive thrombocytopenia, recent heparin exposure, and new thrombotic events. The pharmacist systematically evaluated the patient's clinical course, calculated a 4Ts score of 6 points, recommended HIT antibody testing, and advised immediate discontinuation of enoxaparin and initiation of a non-heparin anticoagulant. Based on this recommendation, enoxaparin was discontinued, and fondaparinux sodium was initiated at 5 mg subcutaneously once daily. The detailed components and scoring process of the 4Ts assessment are shown in Table 1. The high probability 4Ts score (6 points) prompted immediate discontinuation of enoxaparin and initiation of a non-heparin anticoagulant while awaiting laboratory confirmation.

**Table 1:** Detailed assessment of the 4Ts score for suspected heparin-induced thrombocytopenia.

4Ts score component	2 points	1 point	0 points	Patient assessment	Score
Thrombocytopenia	Platelet count fall >50% and nadir ≥20 × 10^9^/L	Platelet count fall 30%–50% or nadir 10–19 × 10^9^/L	Platelet count fall <30% or nadir <10 × 10^9^/L	Platelet count decreased from 109 × 10^9^/L to 27 × 10^9^/L (>50% fall; nadir ≥20 × 10^9^/L)	2
Timing of platelet count fall	Clear onset between days 5–10 after heparin exposure or fall ≤1 day with heparin exposure within previous 30 days	Consistent with days 5–10 fall but unclear; onset after day 10; or fall ≤1 day with heparin exposure 30–100 days earlier	Platelet fall <4 days without recent heparin exposure	Platelet count began to decline approximately 5–7 days after initiation of heparin exposure	2
Thrombosis or other sequelae	New thrombosis or skin necrosis; acute systemic reaction after IV heparin bolus	Progressive/recurrent thrombosis; non-necrotizing skin lesions; suspected thrombosis	None	New bilateral calf intermuscular venous thrombosis and pulmonary embolism were confirmed	2
Other causes of thrombocytopenia	None apparent	Possible	Definite	No alternative cause was identified after clinical evaluation	0
Total score				High probability of HIT	6

On January 1, 2026, the platelet counts further decreased to 27 × 10^9^/L, anti-PF4/heparin antibody testing returned positive, with a quantitative antibody level of 5.7 U/mL (reference range, 0–1.0 U/mL). The assay was performed using a commercially available kit (Werfen, Bedford, MA, USA) that detects mixed antibodies (IgG/IgA/IgM). Optical density values were not routinely reported by clinical laboratories. Functional assays, including the Serotonin Release Assay (SRA) and Heparin-Induced Platelet Activation (HIPA) test, were unavailable at our institution and therefore were not performed. Considering the high score of 4Ts, new thrombotic events, significant platelet decline after heparin exposure, and positive anti-PF4/heparin antibody test results, a diagnosis of HITT was established. Fondaparinux sodium was continued, and the platelet count, coagulation parameters, and clinical symptoms were closely monitored. The sequential changes in platelet count, D-dimer level, anticoagulant therapy, and major clinical events are summarized in Table 2.

**Table 2:** Summary of clinical course, anticoagulant management, laboratory changes, and major clinical events during hospitalization and follow-up.

Date	Platelet	D-dimer	Anticoagulant	Event
December 24, 2025	109 × 10^9^/L	25.55 mg/L	Enoxaparin 3,000 IU qd	Fall-related femoral fracture
December 26, 2025	/	7.55 mg/L	Enoxaparin 2,000 IU qd after surgery	Right hip hemiarthroplasty
December 27, 2025	86 × 10^9^/L	3.53 mg/L	Apixaban 2.5 mg bid	Postoperative thromboprophylaxis after surgery
December 30, 2025	116 × 10^9^/L	7.86 mg/L	Enoxaparin 4,000 IU q12h	Bilateral calf intermuscular venous thrombosis; Multiple segmental and subsegmental pulmonary emboli
December 31, 2025	60 × 10^9^/L	4.40 mg/L	Fondaparinux 5 mg qd	Suspect HIT
January 1, 2026	27 × 10^9^/L	8.62 mg/L	Fondaparinux 5 mg qd	Diagnosed with HITT
January 6, 2026	40 × 10^9^/L	8.16 mg/L	Fondaparinux 5 mg qd	HITT therapy
January 8, 2026	80 × 10^9^/L	4.99 mg/L	Fondaparinux 5 mg qd	Transferred to a local hospital
January 16, 2026	253 × 10⁹/L	/	Anticoagulant therapy had been discontinued after discharge from the local hospital	No recurrent thrombosis or bleeding events

Platelet counts gradually recovered over the following days. On January 6, the platelet count increased to 40 × 10^9^/L, although the D-dimer level remained elevated at 8.16 mg/L. The multidisciplinary team continued the fondaparinux therapy. Given the improvement in platelet count, stable hemoglobin levels, absence of bleeding manifestations, and body weight-based dosing recommendations, standard-dose anticoagulation with fondaparinux sodium (7.5 mg subcutaneously once daily) was recommended after transfer to a local hospital. The exact dose administered after the transfer could not be confirmed. Considering the need for long-term anticoagulation, a transition to warfarin with international normalized ratio-guided dose adjustment is recommended when clinically appropriate.

The patient was transferred to a local hospital on January 8, 2026, with a platelet count of 80 × 10^9^/L and D-dimer level of 4.99 mg/L. No new thrombotic events or bleeding complications were observed prior to transfer. At outpatient follow-up approximately one week later (January 16, 2026), the platelet count had recovered to the normal range (253 × 10^9^/L), and hemoglobin levels had also returned to normal. During subsequent telephone follow-up with the clinical pharmacist, the patient reported no recurrent thrombosis or bleeding events. She reported that anticoagulant therapy had been discontinued after discharge from the local hospital, without medical supervision. The pharmacist advised timely clinical reassessment and emphasized the importance of appropriate, long-term anticoagulant management.

## 3 Discussion

HITT is an uncommon but potentially life-threatening, immune-mediated complication of heparin exposure. Unlike previously reported HIT cases that mainly focus on diagnostic confirmation or anticoagulant selection, the present case highlights several clinically relevant challenges, including distinguishing HIT from common postoperative thrombocytopenia after orthopedic surgery, recognizing HIT in the setting of new thrombotic events, and optimizing anticoagulant therapy through multidisciplinary collaboration. In this case, clinical pharmacist intervention contributed to the early recognition of HIT, timely diagnostic evaluation, and individualized anticoagulant adjustment in an older patient with multiple thrombotic complications.

Patients undergoing major orthopedic surgery represent a particularly challenging population for HIT diagnosis because they have both a high baseline risk of VTE and frequent exposure to heparin-based thromboprophylaxis. Hip fracture surgery and joint replacement procedures are associated with substantial thrombotic risks, and pharmacological prophylaxis is routinely recommended (4, 6). However, postoperative thrombocytopenia is common and may result from multiple factors, including surgical blood loss, hemodilution, infection, inflammatory responses, and medication. Therefore, early recognition of HIT requires careful assessment of the timing of platelet decline, recent heparin exposure, thrombotic manifestations and laboratory findings. Current diagnostic strategies emphasize the integration of clinical probability assessment, particularly the 4Ts score, with anti-PF4/heparin antibody testing to guide diagnosis and management (8–11).

**Figure 1:**
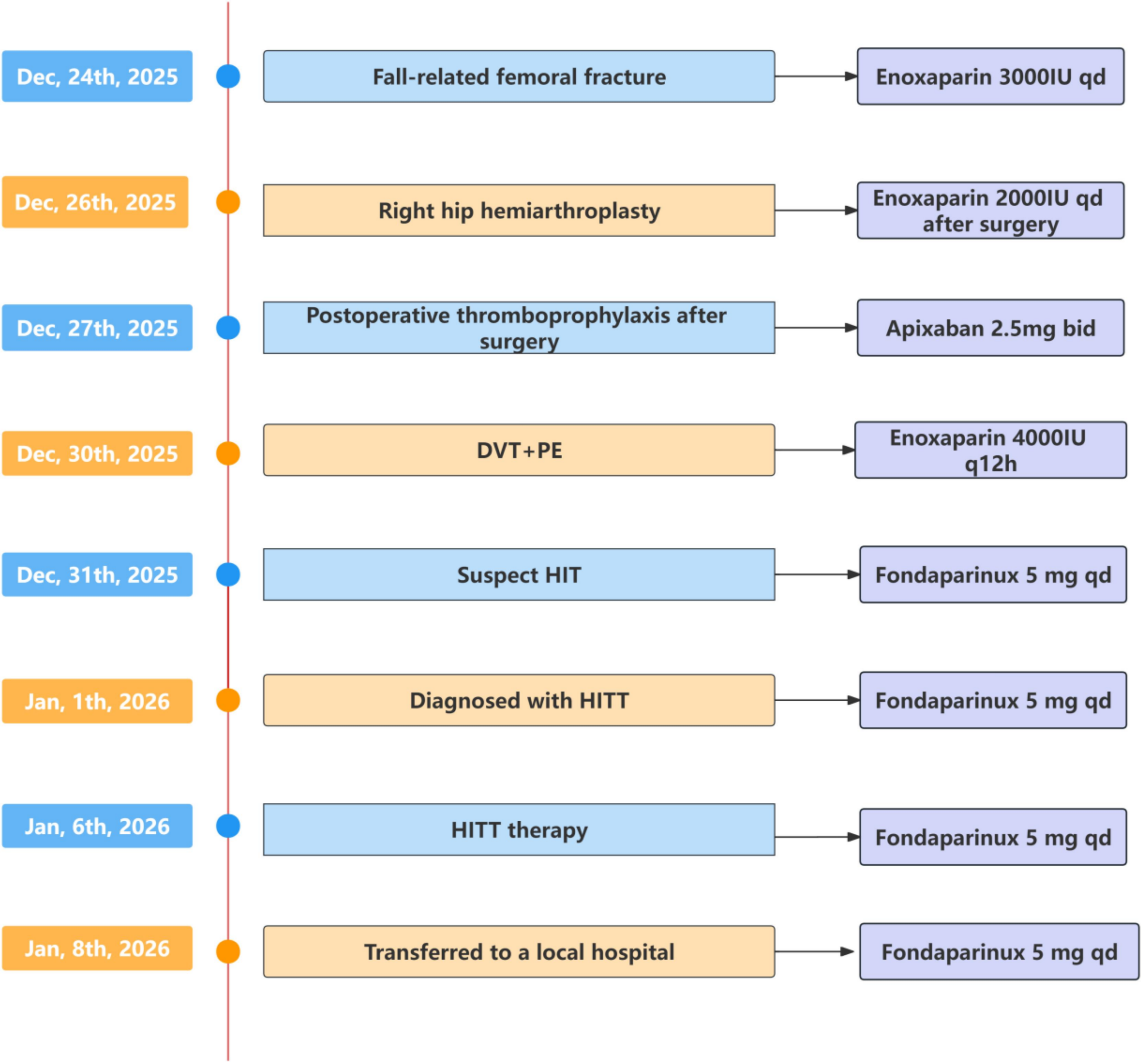
Clinical timeline of the patient's hospitalization and management of HITT.

Previous reports have described HIT occurring after orthopedic surgery, including hip fracture surgery, often presenting with thrombotic complications and diagnostic challenges (5, 7). Similar to these reports, our patient developed HITT following hip hemiarthroplasty, with progressive thrombocytopenia accompanied by bilateral lower extremity thrombosis and pulmonary embolism. However, this case further highlights the importance of continuous clinical reassessment during postoperative anticoagulant therapy. At the time, therapeutic enoxaparin was initiated for acute thromboembolism, and HIT was not suspected. Following clinical pharmacist consultation, the patient's platelet trend, timing of heparin exposure, and new thrombotic events were systematically evaluated, resulting in a high probability 4T's score and subsequent HIT antibody testing. The major clinical events, anticoagulant changes, and diagnostic milestones are summarized in Figure 1. This case emphasizes that postoperative thrombocytopenia should not be automatically attributed to surgical factors, particularly when accompanied by new thrombotic manifestations.

The cornerstone of HIT management is the immediate discontinuation of all heparin-containing products and initiation of alternative anticoagulation therapy (2, 3). Available non-heparin anticoagulants include direct thrombin inhibitors, fondaparinux, and direct oral anticoagulants; however, evidence supporting the optimal choice of anticoagulant remains limited, and treatment decisions should be individualized according to patient characteristics, bleeding risk, renal function, drug availability, and monitoring requirements (2, 3, 10–12). In this case, fondaparinux was selected after a multidisciplinary evaluation because it does not form immune complexes with PF4/heparin antibodies, has predictable pharmacokinetic properties, and allows once-daily subcutaneous administration (13). Before initiation, renal function was carefully assessed, with a serum creatinine level of 42.9 μmol/L and an estimated creatinine clearance of 66.61 mL/min, which supported its use in this patient. Considering the patient's advanced age, low body weight, and risk of postoperative bleeding, an individualized initial dosing strategy was adopted. As the platelet count recovered, hemoglobin levels remained stable, and no bleeding manifestations occurred, standard weight-based anticoagulation with fondaparinux sodium was subsequently recommended.

The role of clinical pharmacists is an important aspect of this case. Pharmacists' involvement in anticoagulation management has expanded beyond medication review to include risk assessment, therapeutic monitoring, and optimization of complex anticoagulant regimens. In the present case, the clinical pharmacist identified the possibility of HIT based on the patient's clinical trajectory, calculated the 4Ts score, recommended HIT antibody testing, advised discontinuation of enoxaparin, and recommended initiation of fondaparinux. These recommendations were accepted by the treating physicians and contributed to the timely modification of anticoagulant therapy and avoidance of continued heparin exposure. Furthermore, pharmacist follow-up after discharge identified that the patient had discontinued anticoagulant therapy without medical supervision, reinforcing the need for timely reassessment and appropriate long-term anticoagulant management.

For patients with HIT complicated by thrombosis, therapeutic anticoagulation should generally be continued for an appropriate duration to reduce the risk of recurrent thromboembolic events, with the treatment duration individualized according to the thrombotic burden and clinical recovery (14, 15). In this case, the patient achieved platelet recovery without recurrent thrombosis or bleeding during the follow-up period. However, long-term management was limited by the transfer of care to another hospital and incomplete outpatient follow-up. This study has several limitations. First, functional HIT assays, including the serotonin release assay and heparin-induced platelet activation test, were unavailable at our institution; therefore, the diagnosis was based on clinical probability assessment and anti-PF4/heparin antibody testing. Second, as a single case report, this study cannot establish the superiority of a specific anticoagulant strategy over others. Nevertheless, this case provides practical insights into the recognition and management of HITT after orthopedic surgery and highlights the potential contribution of clinical pharmacists to multidisciplinary anticoagulation care.

## 4 Conclusion

HITT is a potentially life-threatening complication that may be difficult to recognize after orthopedic surgery because of the multiple possible causes of postoperative thrombocytopenia. This case highlights the importance of continuous platelet monitoring, timely assessment of HIT probability, and prompt adjustment of anticoagulant therapy when new thrombotic events occur. Clinical pharmacist involvement contributed to the early recognition of HIT, calculation of the 4Ts score, initiation of appropriate diagnostic evaluation, and optimization of non-heparin anticoagulant therapy. Individualized management with fondaparinux, guided by the patient's thrombotic risk, bleeding risk, body weight, and renal function, was associated with platelet recovery without recurrent thrombosis or bleeding during the follow-up period. This case emphasizes the value of multidisciplinary collaboration in the management of HIT after orthopedic surgery and suggests that clinical pharmacists may play an important role in improving anticoagulation decision-making, medication monitoring, and patient safety in complex clinical settings.

## Data Availability

The original contributions presented in the study are included in the article/Supplementary Material, further inquiries can be directed to the corresponding author.
